# New lunar crater reveals extensive distal regolith modification

**DOI:** 10.1126/sciadv.aeh9568

**Published:** 2026-09-16

**Authors:** Tyler M. Powell, Benjamin T. Greenhagen, Tyler Horvath, Jean-Pierre Williams, Elisha Jhoti, Emerson J. Speyerer, Catherine M. Elder, David A. Paige

**Affiliations:** ^1^ Johns Hopkins University Applied Physics Laboratory, Laurel, MD, USA.; ^2^ Department of Earth, Planetary, and Space Sciences, University of California, Los Angeles, CA, USA.; ^3^ Intuitive Machines, Phoenix, AZ, USA.; ^4^ Jet Propulsion Laboratory, California Institute of Technology, Pasadena, CA, USA.

## Abstract

A new ∼222-meter-diameter impact crater formed on the Moon in spring 2024. Discovered in repeat Lunar Reconnaissance Orbiter Camera images, it provides a unique opportunity to study surface modification by a pristine impact, prior to subsequent fading. Nighttime temperatures measured by the Diviner instrument reveal an ∼7-kilometer-wide cold spot that is 8 to 9 kelvin cooler than its surroundings, consistent with decompaction of the upper centimeters to decimeters of regolith. We investigate all newly formed craters >20 meters identified to date and find that all craters larger than ∼30 meters produce detectable cold spots, indicating that cold spot formation is a common consequence of fresh lunar impacts. Cold spot temperature anomaly increases with crater size, implying deeper regolith modification around larger craters. This scaling suggests that the cumulative effect of cold spot formation may modify the shallow regolith at rates comparable to impact gardening by primary craters, making them a substantial driver of regolith evolution on regolith-covered airless bodies.

## INTRODUCTION

A new ∼222-m-diameter impact crater (McGetchin crater) formed on the Moon in spring 2024, discovered in repeat Lunar Reconnaissance Orbiter Camera (LROC) images (*1*, *2*). It is by far the largest fresh crater identified since the mission began in 2009, exceeding the previous largest at 70 m (*3*, *4*). Crater production models predict that an impact of this size occurs on the Moon only once every ∼132 years on average (*5*), making it an event of century-scale rarity. McGetchin crater’s size and pristine state make it an exceptional natural experiment for observing the initial response of the lunar surface to a fresh impact, prior to subsequent alteration by space weathering, impact gardening, and other surface processes.

This opportunity is especially important for understanding lunar cold spots, first discovered in nighttime temperature data from the Diviner Lunar Radiometer onboard the Lunar Reconnaissance Orbiter (LRO) (*6*, *7*). Cold spots are extensive regions of reduced nighttime temperature relative to surrounding terrain that have been observed around young impact craters between ∼30 m and ∼2 km in diameter. They are enormous relative to their source craters, extending tens to, in some cases, hundreds of crater radii (*6*). They are hypothesized to result from a decompaction of the upper regolith during the impact process; thermal modeling shows that the observed nighttime cooling is consistent with reduced density in the upper centimeters of the regolith, resulting in lower thermal inertia (*6*). This interpretation is supported by in situ measurements obtained during the Apollo 16 mission, which landed on a faint cold spot associated with South Ray crater (*8*). Astronaut footprints were deeper within the South Ray cold spot than at other sites, indicating decompacted regolith (*8*). Cold spots fade on ∼100 ka to ∼2 Ma timescales (*8*, *9*), making them reliable markers of some of the youngest impact craters on the Moon. Their young age has enabled their use in investigations of regolith thickness (*10*, *11*), the recent impact cratering rate (*9*), and as potential source craters for lunar meteorites (*9*). Similar low–thermal inertia anomalies potentially analogous to lunar cold spots have also been observed on Mars (*12*), suggesting that cold spot formation may represent an important process in regolith evolution throughout the solar system, in which even relatively small craters can physically modify the regolith over great distances.

The short lifetime of cold spots complicates efforts to constrain their initial properties, as many present-day cold spots are already substantially degraded, making it difficult to separate the effects of crater size, target properties, impact conditions, and age on their initial state. The new ∼222-m McGetchin crater provides the best opportunity yet to observe a large, virtually unaltered cold spot with well-characterized pre-impact conditions. This will improve our understanding of cold spot formation and provide a calibration for the fading timescale of cold spots, potentially enabling age estimates for some of the youngest craters on the Moon using cold spot properties. Constraints on the magnitude and spatial extent of regolith modification from new cold spot formation also enable estimates of their cumulative contribution to regolith evolution.

In this work, we analyze Diviner measurements of the 21 newly formed craters identified by LROC temporal imaging that are larger than 20 m in diameter (*3*, *4*), including the McGetchin crater (*1*), to assess the occurrence and properties of their cold spots. We define “newly formed craters” as those identified to date by comparing temporal pairs in LROC images (*1*, *3*, *4*). New craters are referred to by diameter-ranked indices (e.g., NC-A or McGetchin crater is 222 m, NC-B is 70 m, etc.) (table S1). We compare their properties to the global population of preexisting cold spots formed before the start of the LRO mission and investigate the implications of cold spot formation on global regolith evolution.

## RESULTS

### Spatial structure of the McGetchin crater cold spot

Diviner began observing McGetchin crater as soon as it was discovered in fall 2025. The first observations were obtained on 1 November 2025, ∼1.5 years after the spring 2024 formation date inferred from LROC images. This was too late after impact to observe a positive thermal anomaly associated with residual heat from the impact event itself. Such an anomaly was observed by Diviner after the LCROSS spacecraft impacted the Moon (*13*); elevated temperatures were detected ∼90 s after impact and on two subsequent orbits, but faded to near-undetectability after ∼4 hours. As of 18 February 2026, McGetchin crater has been observed by Diviner during nine campaigns, each comprising several targeted observations acquired during alternating orbits. The dataset totals 43 orbits, including 20 nighttime orbits used to constrain thermophysical properties (fig. S1).

**Fig. 1.**
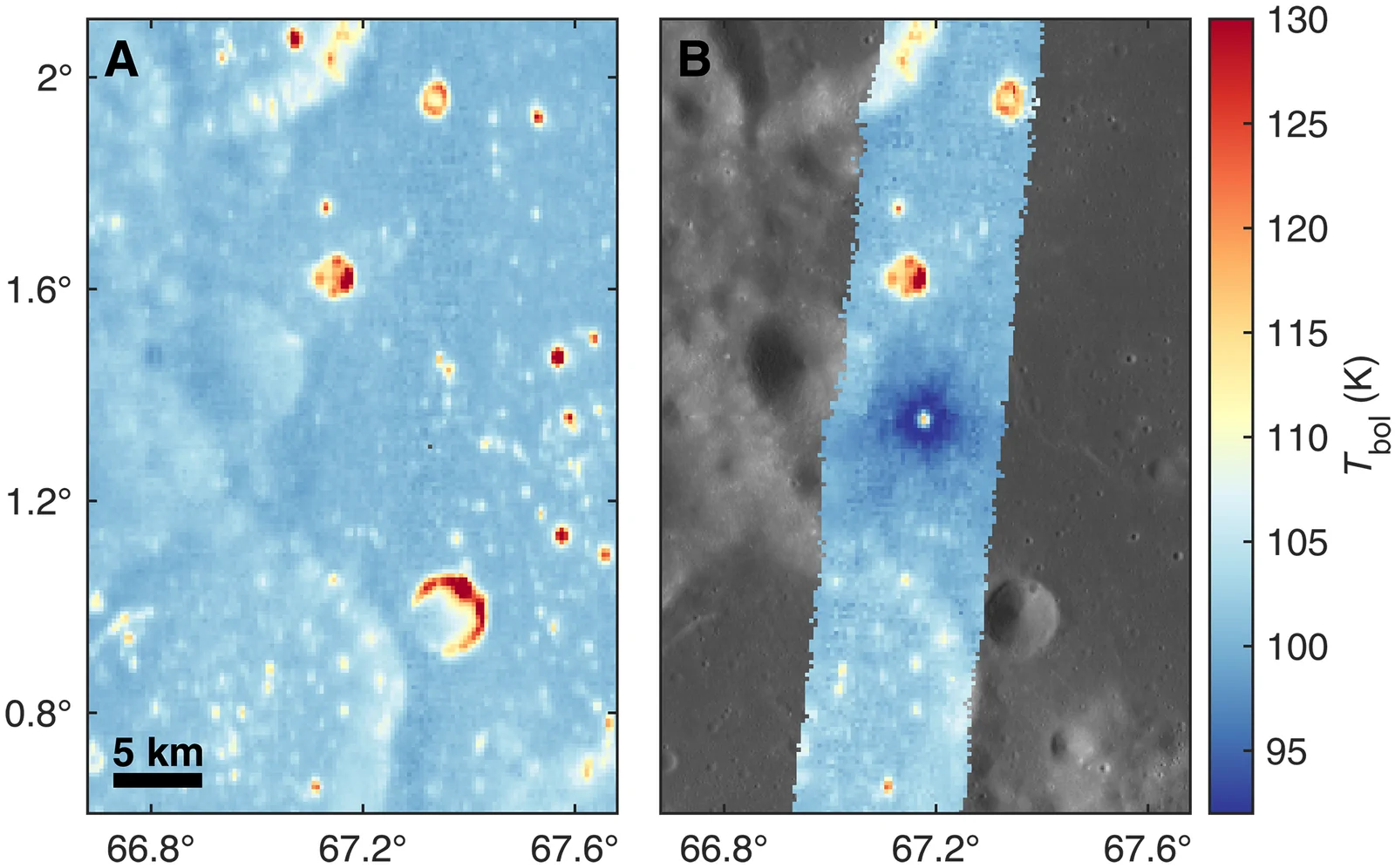
Newly formed cold spot in post-impact nighttime temperature. Diviner bolometric temperature interpolated to local midnight (**A**) pre- and (**B**) post-impact. Pre-impact *T*_bol_ is from the Diviner Global High-Resolution Mosaics (*27*). Post-impact *T*_bol_ is produced using nighttime data collected between 15 November 2025 and 18 February 2026.

Figure 1 shows bolometric temperature interpolated to local midnight before and after impact, revealing a warm, rocky central crater and proximal ejecta surrounded by a prominent ∼7-km-wide cold spot. Several low-temperature rays extend radially from the crater, some spatially coincident with reflectance changes in McGetchin crater’s distal ejecta observed in LROC images (*1*). The most distinct low-temperature ray extends to the north-northeast at an azimuth ∼20° from north, matching the direction of both a tongue of low albedo material in the crater’s proximal ejecta [figure 1 in (*1*)], as well as a prominent ray in the distal low-reflectance zone (DLRZ) [figure 6 in (*1*)]. Although the low-temperature rays align in azimuth with features in both the proximal ejecta and DLRZ, their spatial extents are substantially different. The DLRZ of McGetchin crater shows faint changes in reflectance to distances of ∼100 km (*1*), roughly 30 times the radial extent of the cold spot.

This spatial similarity in shape but difference in size may suggest that cold spots and DLRZs arise from similar processes, but with different scales of modification. Modification to centimeter-scale depths is required to influence nighttime temperatures, whereas visible photometric properties are sensitive to grain-scale alteration (tens of micrometers to submillimeters). Photometric investigations of new-crater DLRZs attribute the reflectance change to increased roughness rather than to changes in composition or intrinsic albedo (*3*, *4*, *14*). Similarly, polarimetric observations of cold spots by the PolCam instrument on the Korea Pathfinder Lunar Orbiter identify a photometric anomaly associated with some cold spots, consistent with increased roughness (*15*).

### Roughness anomaly in Diviner daytime data

**Fig. 2.**
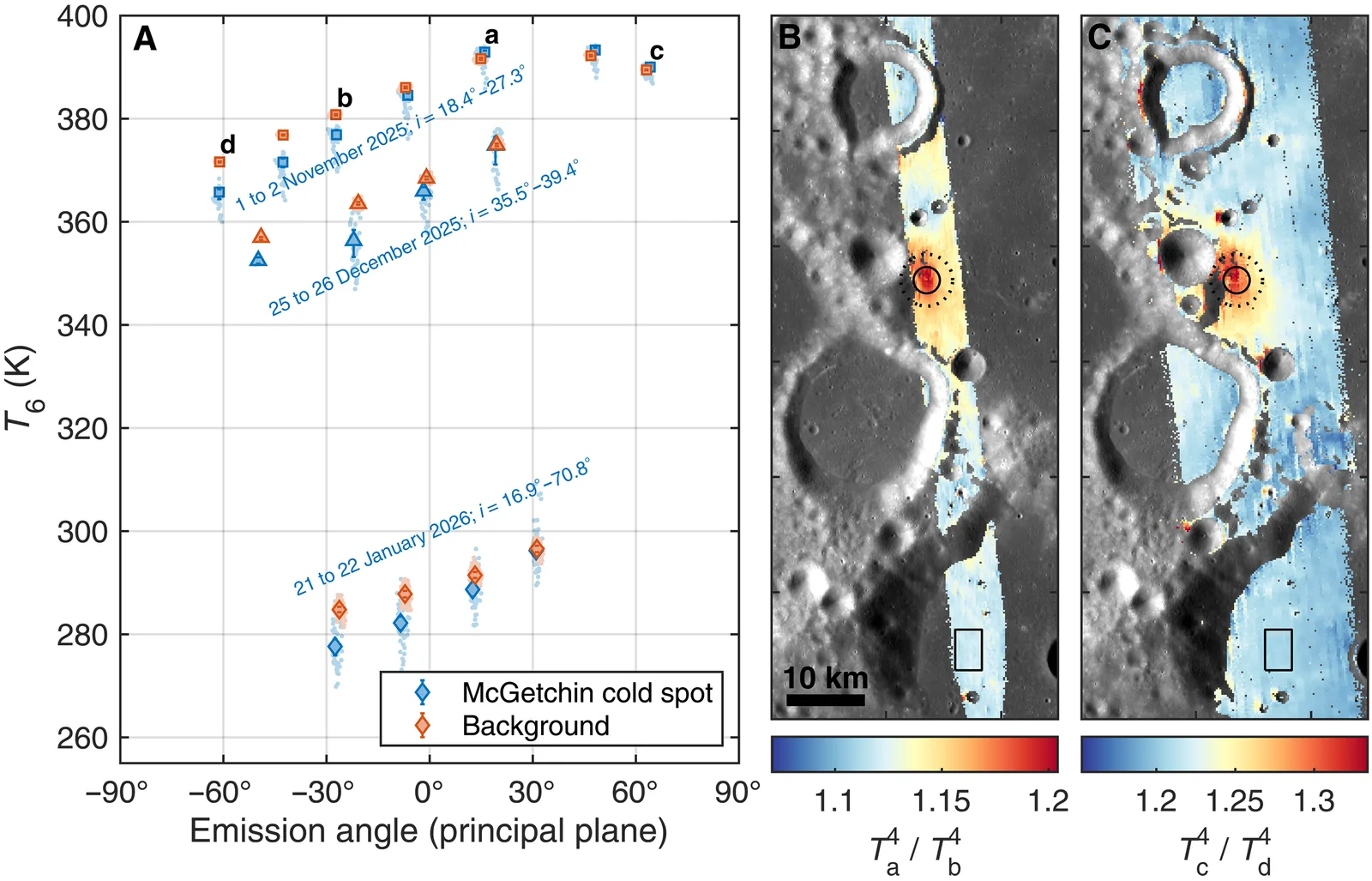
Daytime EPF indicates elevated roughness. (**A**) Diviner Channel 6 brightness temperatures in the principal plane for the McGetchin cold spot and nearby background terrain, collected during three off-nadir targeting campaigns. Positive emission angle denotes viewing geometries where the spacecraft and the Sun are in the same direction, and negative emission angle denotes geometries in which they are in opposing directions. (**B** and **C**) Ratio images for orbits with positive and negative emission angle at similar incidence angle, with slopes >8° excluded. Data in (A) were extracted from the solid circle and rectangle for the cold spot and background, respectively. The black dotted circle is 7 km in diameter, noting maximum extent of the nighttime cold spot.

The Diviner daytime emission phase function (EPF) is likewise sensitive to roughness (*16*–*22*). For illuminated terrain, small-scale roughness results in a range of surface temperatures, primarily driven by the orientation of slopes to the sun, which results in variations in observed brightness temperatures with viewing geometry. Thermal emission probes roughness at millimeter to subcentimeter scales, as thermal gradients over smaller distances are suppressed by conduction (*17*). Post-impact daytime EPF data were acquired during several targeted campaigns in which Diviner pointed off-nadir to the west and east on approach and regress, respectively, providing complementary observations with similar incidence angle but substantially different emission angle. Relative to background terrain, the region surrounding McGetchin crater is consistently warmer when viewed in the pro-sun direction and cooler when viewed from the anti-sun direction, a signature consistent with increased surface roughness (Fig. 2) (*16*, *20*, *21*). This occurs because pro-sun viewing of a rough surface preferentially samples illuminated, warmer subresolution facets, whereas anti-sun viewing preferentially samples poorly illuminated or shadowed facets.

The observed EPF anomaly around McGetchin crater is ∼14 km across, extending to roughly two times the size of the cold spot. This is consistent with EPF being sensitive to millimeter- to subcentimeter-scale surface roughness, while nighttime temperatures require alteration at centimeter-scale depths. Similarly, the EPF anomaly is smaller than the DLRZ in visible images [figure 6 in (*1*)]. Grain-scale surface roughening would not be detected in thermal emission but could be detected in faint differences in pre- and post-impact visible photometry. High–phase angle PolCam images reveal dark halos around fresh craters. These are smaller than cold spots, extending ∼2 to 3 crater radii, and have been attributed to substantial roughening within the proximal ejecta (*23*). The proximal dark halos may form through processes distinct from, but potentially related to, those that produce the more distal cold spots and DLRZs. Although photometry can be sensitive to fine-scale surface texture, the observed dark halos are associated with high thermal inertia and are visibly rocky and morphologically distinctive in NAC images (*23*), suggesting that they may also include a coarser roughness component within the proximal ejecta. These impact-related phenomena suggest a consistent trend of increasing radial extent with sensitivity to smaller-scale disturbances.

Similarly, the retention age of these features likely also shows a trend of finer-scale disturbances fading on shorter timescales. Repeat LROC images of the 70-m crater NC-B since its formation in 2012 show measurable fading of the DLRZ, and extrapolation suggests that it may disappear entirely on the order of ∼50 years (*24*). This is shorter than the lifetime of cold spots, which fade on ∼100 ka to Ma timescales (*8*, *9*). The PolCam high–phase angle dark halos fade on timescales of ∼3 to 4 Ma (*23*), consistent with the trend of roughening closer to the impact site re-equilibrating on longer timescales. Although the fading timescale of EPF anomalies remains unconstrained, we do not observe a prominent EPF anomaly around South Ray crater, suggesting that EPF anomalies fade on shorter timescales than cold spots. Future work quantifying the occurrence of EPF anomalies around cold spots and continued Diviner monitoring of McGetchin crater will help constrain the retention timescale of these anomalies.

### Geologic context

McGetchin crater formed extremely close to the boundary between an unnamed mare deposit and the proximal ejecta of highlands crater Dubyago N (*1*), with the cold spot extending across both mare and highlands terrain. Despite this complex geologic context, the cold spot is approximately symmetrical, with little difference in temperature anomaly between the highlands to the west and mare to the east. This symmetry indicates that cold spots form similarly in mare and highlands terrain, likely because the upper several centimeters of regolith that control nighttime temperatures have similar thermophysical properties across both the mare and highlands, as is typically observed globally for intercrater terrain (*25*–*27*). This interpretation is consistent with other cold spots located near terrain boundaries, which also show little evidence for terrain-related asymmetry.

**Fig. 3.**
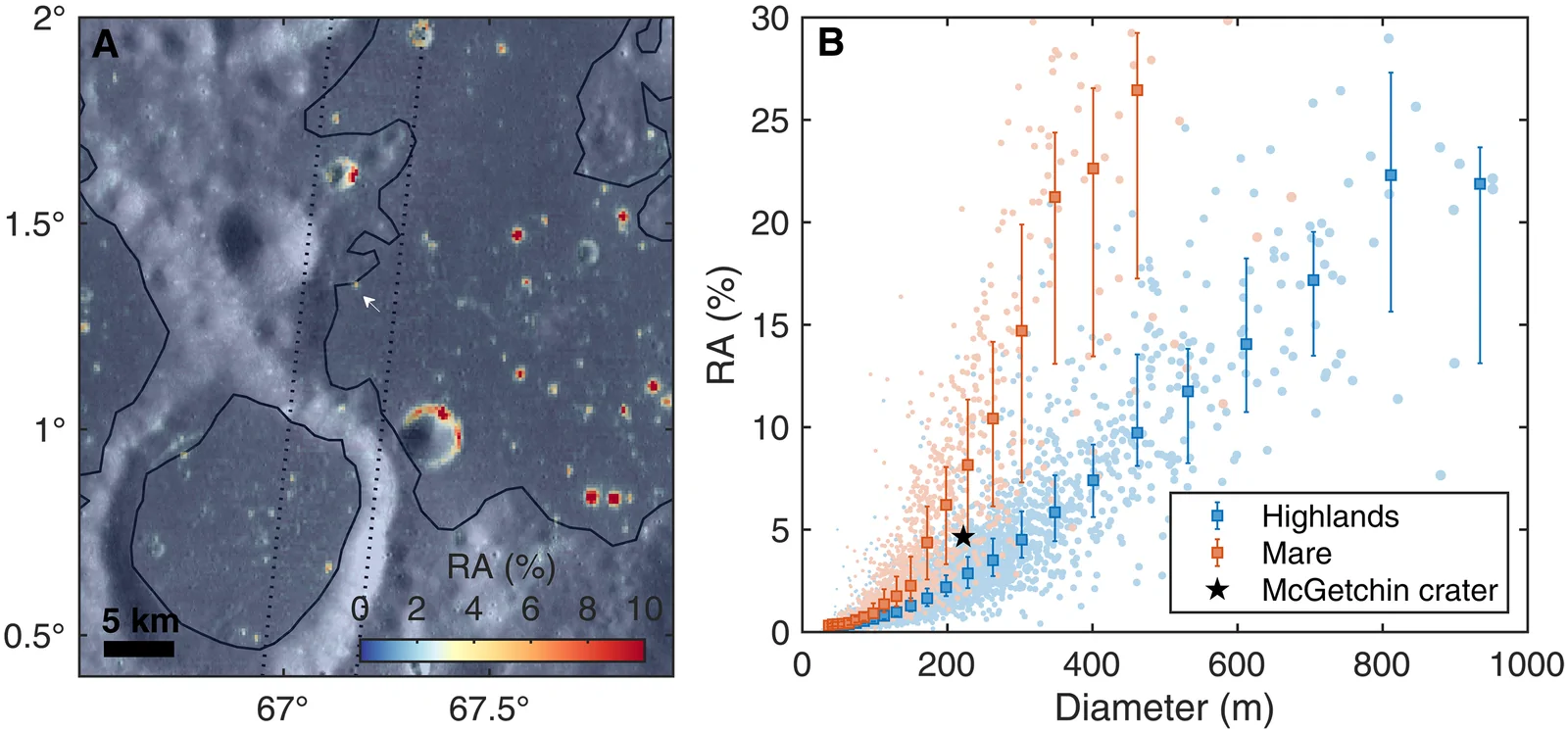
McGetchin crater RA is low for a mare crater. (**A**) Combined post-impact RA where available (dotted outline) with the pre-impact global RA map (*27*) elsewhere. Black lines show the highlands/mare boundary defined by (*51*). (**B**) Maximum RA of cold spot craters globally as a function of crater diameter. The RA of McGetchin crater is intermediate between highlands and mare.

Post-impact Diviner rock abundance (RA) for McGetchin crater shows a rocky central crater, a feature that is not resolved for the other, smaller new craters. The crater has a peak RA of 4.64%, which is lower than the typical value for similarly sized mare cold spot craters [8.16%, interquartile range (IQR): 4.38 to 11.35%], but higher than that of highlands cold spot craters (2.86%, IQR: 2.15 to 3.67%) (Fig. 3). Low-reflectance material in McGetchin crater’s proximal ejecta seen in LROC images is consistent with a mixture of highlands and mare material, suggesting that the crater’s position on or near the surface expression of the mare/highlands contact allowed it to sample both terrains [figure 8 in (*1*)]. This intermediate RA supports that interpretation and is consistent with either penetration through a thin mare layer into underlying rock-poor regolith or excavation from a transitional boundary zone. A thin mare layer may also limit the size and abundance of rocks in the ejecta, as lava flows only a few meters thick may produce fewer meter-scale blocks than thicker flows (*28*).

### Comparison of cold spot intensity

**Fig. 4.**
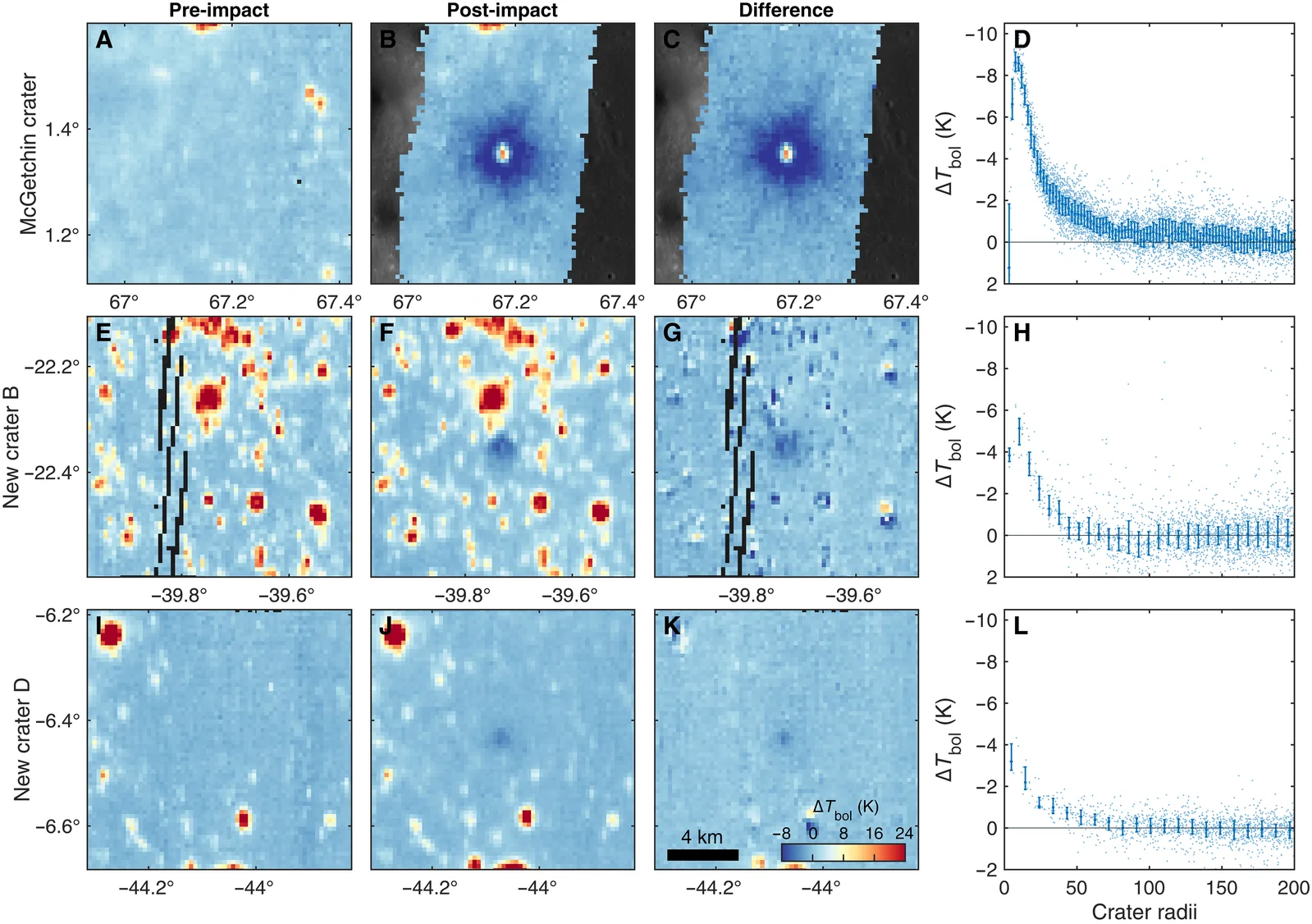
Radial profile of temperature anomaly for newly formed cold spots. Examples of pre-impact (**A**, **E**, and **I**) and post-impact (**B**, **F**, and **J**) ∆*T*_bol_, and post-impact–minus–pre-impact difference (**C**, **G**, and **K**) for three new craters with identifiable cold spots. Radial profiles (**D**, **H**, and **L**) show the median and interquartile range of the ∆*T*_bol_ difference in 240-m bins.

Figure 4 shows pre- and post-impact temperature anomaly for three representative new cold spots, formed after the start of the LRO mission in 2009 (see Materials and Methods). McGetchin crater (NC-A) exhibits a peak cold spot temperature anomaly, ∆*T*_bol_, of −8.61 K, while the smaller NC-B and NC-D show weaker anomalies of −5.14 and −3.20 K, respectively. All new craters larger than 30 m have detectable cold spots (fig. S2). In the 20- to 30-m diameter range, some craters exhibit detectable cold spots, while others do not or are difficult to identify. Cold spots may still form around these smaller craters but may be too small or faint to detect at the current resolution. However, the absence of detection cannot be explained by resolution alone. Modeling of Diviner’s spatial response (fig. S3) (*27*, *29*) indicates that a 20-m crater should produce a cold spot ∼3 Diviner pixels across, with a measured temperature anomaly ∼40% of its true peak value. This occurs because each pixel measures a mixture of emission from cold spot regions with adjacent weaker or non–cold spot regions. For a detection cutoff of ∼0.5 K, small cold spots must have amplitudes of <1 K to escape identification. The decline in cold spot intensity and detectability at small sizes persists even after accounting for resolution effects, indicating that the cold spots around smaller craters are intrinsically weaker and not simply more difficult to resolve. The presence of cold spots around all new craters large enough for reliable detection indicates that cold spots are ubiquitous and likely form around all lunar craters larger than ∼30 m in diameter.

**Fig. 5.**
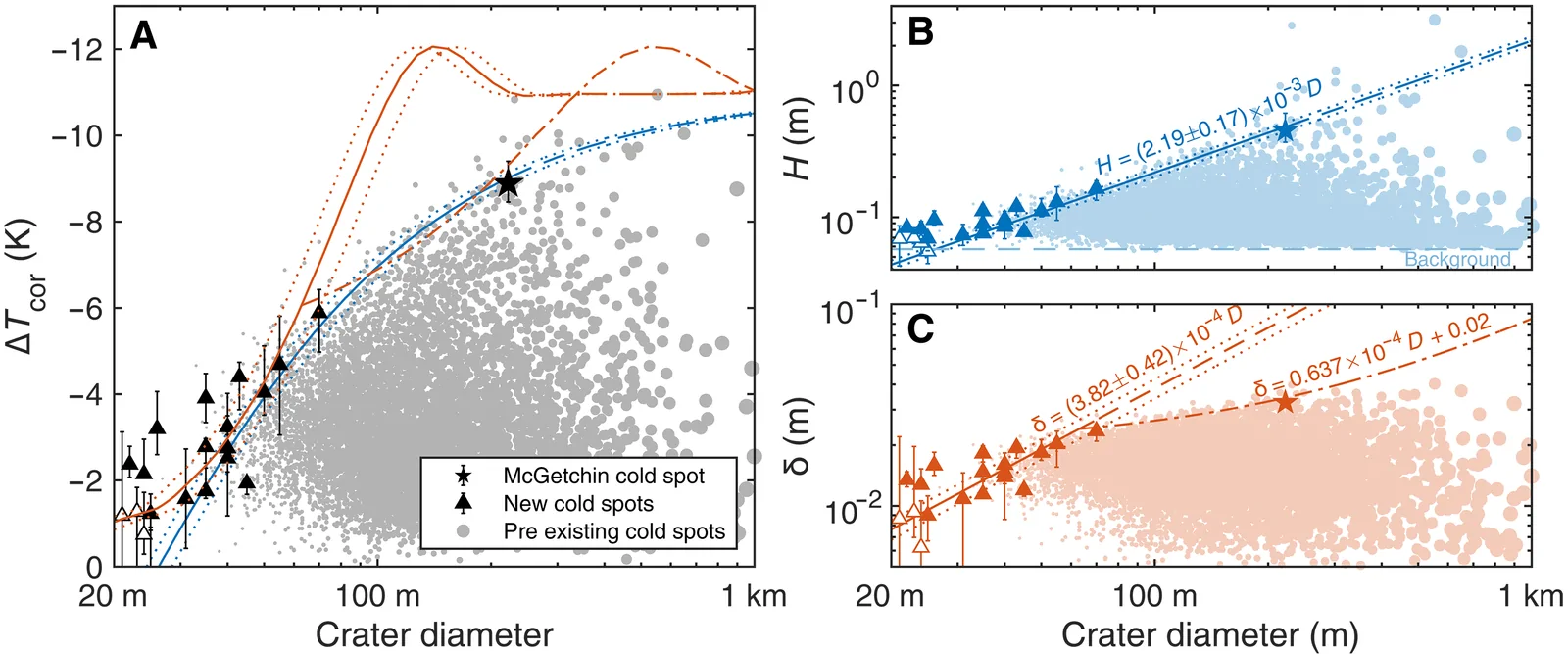
Scaling of cold spot properties. (**A**) Peak ∆*T*_cor_ for new and preexisting cold spots. The white triangular markers indicate new craters with uncertain cold spot detections. New cold spots form an upper envelope to the properties of the entire cold spot population. (**B** and **C**) Equivalent peak *H* and δ values determined using a thermal model. The blue and red curves show linear best-fits to the H- and δ-models, respectively. In (A), these linear fits are expressed in terms of their equivalent temperature anomaly. The dash-dotted red line corresponds to a flattening of the δ-model for δ > 2.4 cm, which better matches the shape of the cold spot property envelope.

Figure 5A shows the resolution-corrected peak temperature anomaly, ∆*T*_cor_, of new cold spots as a function of crater diameter, compared with a global catalog of preexisting cold spots (fig. S4) (see Materials and Methods). The ∆*T*_cor_ of new cold spots scales strongly with crater diameter. Additionally, the new cold spots are consistently among the most prominent of their size on the Moon, and their ∆*T*_cor_ values define an upper envelope to the preexisting population. The consistency of this relationship suggests that new cold spots form with predictable initial intensity, and we see little evidence to suggest that variations in target properties or impact conditions substantially influence the state of the thermal anomalies. Although there is some scatter in the ∆*T*_cor_ of new cold spots possibly related to impact or target properties, preexisting cold spots generally do not exceed new cold spots, despite the global catalog spanning the full range of lunar terrain types. This likely reflects the broadly uniform thermophysical properties of the upper tens of centimeters of regolith at ∼100-m lateral scales across much of the Moon (*25*–*27*), which would promote similar cold spot formation across otherwise diverse geologic settings. This also suggests that the present-day variability in cold spot intensity is controlled primarily by age, with cold spots fading from an initial upper envelope over time. Fading of the cold spot temperature anomaly with time has been observed for several larger cold spots dated by crater counting (*8*, *9*) and suggests that cold spot intensity may serve as a useful metric for estimating the ages of some of the youngest lunar craters.

The increase in ∆*T*_cor_ with crater diameter suggests that the larger impacts produce cold spots with more substantial regolith modification. This is broadly consistent with the expectation that larger craters generate approximately self-similar cold spots, with the magnitude of regolith modification scaling with crater size. However, the upper envelope of ∆*T*_cor_ does not continue linearly to larger sizes and instead begins to flatten for craters larger than ∼100 m. This flattening may indicate a true plateau in regolith alteration. Alternatively, regolith modification may continue to increase with crater size without producing a proportional increase in temperature anomaly. Nighttime surface temperatures are most sensitive to thermophysical properties within a shallow layer spanning a few multiples of the thermal skin depth (*z*_skin_ ≈ 4 to 10 cm) (*26*), such that deeper material has progressively less influence on how the surface temperature evolves over the course of the night. Larger craters may produce cold spots with deeper regolith modification, but with diminishing effects on surface temperature. We investigate the position and shape of this rollover using thermal modeling.

### Regolith stratigraphy from thermal modeling

**Fig. 6.**
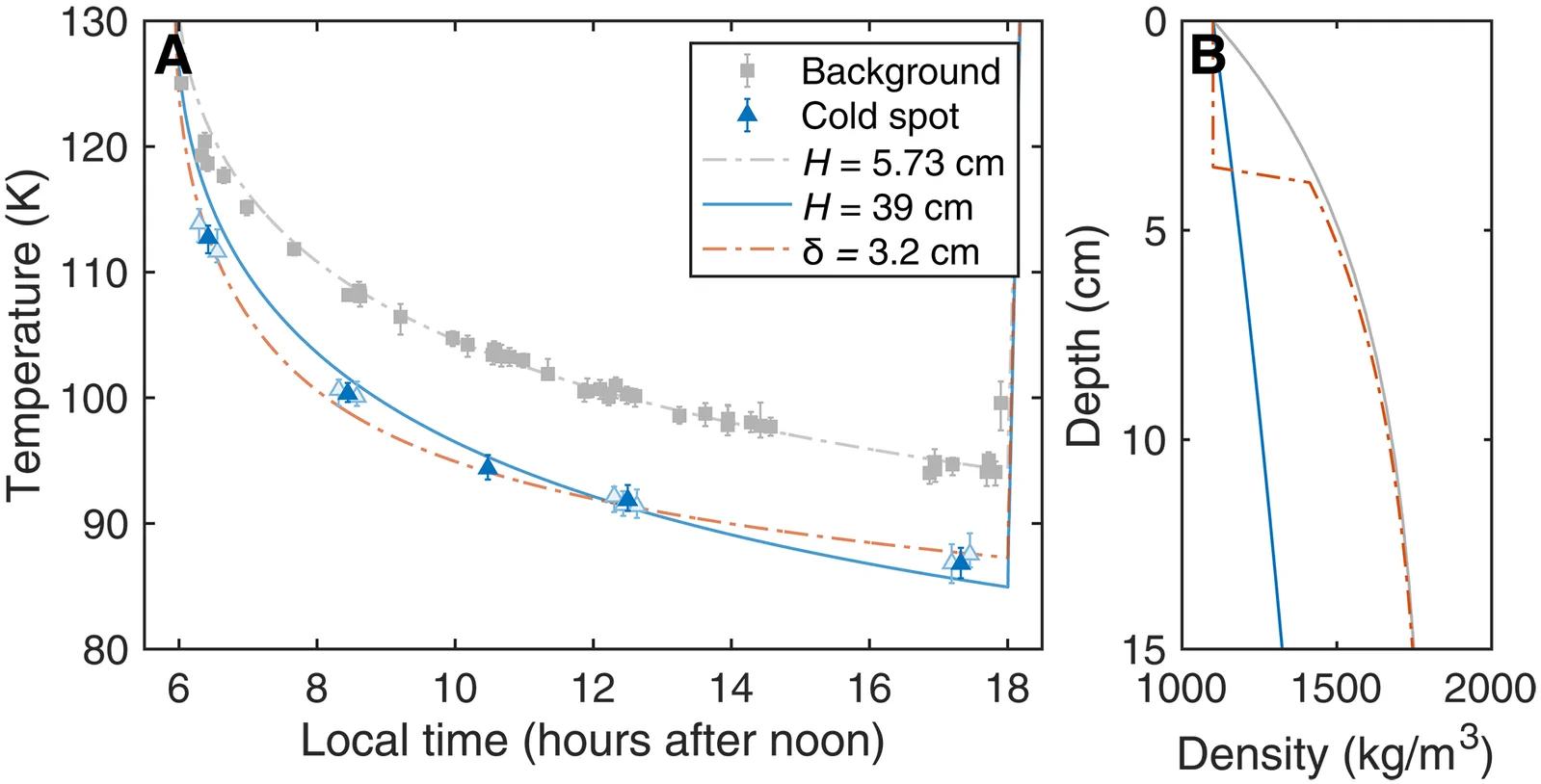
Cooling behavior of the McGetchin crater cold spot. (**A**) Median and interquartile range of *T*_bol_ throughout the night for the cold spot (blue) and typical surrounding regolith (gray) fit using a thermal model. (**B**) The subsurface density profiles for the best-fit thermal models.

Figure 6 shows the nighttime cooling curve of the McGetchin crater cold spot compared with pre-impact regolith at the same location. We fit these temperatures using two end-member subsurface density profiles that bracket the plausible depth of regolith modification. The first assumes an exponential increase in density with depth, which reproduces Diviner nighttime temperatures well for typical regolith (*26*, *30*)

$$\rho =\rho_{d}-\left(\rho_{d}-\rho_{s}\right)e^{-z/H}$$ (1)

where ρ_s_ and ρ_d_ are the densities at the surface and depth, respectively, and the H-parameter, *H*, is the exponential scale height of the lower-density surface regolith layer (Fig. 6B). We refer to this as the “H-model.” We also investigate a two-layer model in which the upper regolith is decompacted to create a uniform low-density layer of thickness δ. Beneath this layer, the density follows the profile of typical regolith with an *H* of 6 cm. We refer to this as the “δ-model.” These two model types define end-members in subsurface structure: The H-model represents a gradual increase in density with depth, whereas the δ-model represents a discrete low-density layer. For both models, we use the heat capacity and thermal conductivity from (*26*), which are broadly consistent with in situ estimates of thermal conductivity from the Apollo heat flow probes (*31*) and the Chandra’s Surface Thermophysical Experiment (ChaSTE) onboard the Chandrayaan 3 lander (*32*).

Using the H-model, the cooling trend of the McGetchin cold spot requires an *H* of ∼39 cm, substantially greater than that of surrounding non–cold spot regolith (∼6 cm). Converting the ∆*T*_bol_ of new cold spots to equivalent *H* values shows that the upper envelope of cold spot properties follows an approximately proportional increase in *H* with crater diameter (Fig. 5B). This trend is consistent with cold spot modification depth scaling linearly with crater diameter and producing a plateau in ∆*T*_bol_ once *H* reaches ∼20 to 30 cm, a few multiples of the diurnal thermal skin depth, and the mean density within this layer asymptotically approaches a minimum value. The model also reproduces the onset of cold spot detection near ∼30-m crater diameter: Smaller craters are expected to produce cold spots with H-parameters of <6 cm, which is the typical value for background regolith. Under this interpretation, these craters may still decompact the regolith, but no noticeable change is observed because the upper ∼6 cm is already decompacted.

Using the δ-model, the McGetchin cold spot is fit by a δ of ∼3.2 cm, much shallower than implied by the H-model, indicating that the anomaly can be reproduced by shallow near-surface decompaction. However, proportional scaling of δ with crater diameter fails to reproduce the observed cold spot trend: a linear fit for new craters <70 m rapidly overshoots the cold spot ∆*T*_bol_ at larger sizes (Fig. 5A). This is made unambiguous by McGetchin crater, the only new crater larger than the rollover diameter, which falls well below the δ-model linear scaling prediction. This disagreement may arise because the δ-model reproduces a given ∆*T*_bol_ with shallower regolith modification, so the best fit-scaling at small sizes (<70 m) does not allow δ to reach the skin depth for ∼100-m craters, shifting the rollover to larger diameters. If regolith modification depth scales proportionately with crater size, the position of the ∆*T*_bol_ rollover suggests that regolith decompaction reaches depths on the order of the thermal skin depth for ∼100-m craters, a behavior not reproduced by the δ-model.

Alternatively, the depth of regolith modification may not continue to scale at the same rate for larger impacts due to regolith properties. Regolith density and mechanical strength increase with depth over the upper several centimeters (*33*), which may make it more difficult to decompact dense material below the looser upper centimeters, and result in a flattening of δ and the resulting ∆*T*_bol_ trend past that depth. We find that a decrease in the scaling of δ with crater diameter by a factor of ∼1/6 for craters larger than ∼50 to 60 m is required to match the upper envelope of cold spot properties, corresponding to an inflection for δ larger than ∼2.4 cm (Fig. 5).

The regolith density profile can also be inferred from the shape of the nighttime cooling curve. Neither the H- nor δ-model perfectly matches the nighttime cooling behavior of the McGetchin cold spot (Fig. 6). It exhibits lower nighttime temperatures early in the night than the H-model predicts. The δ-model, which has lower thermal inertia near the surface, achieves lower temperatures early in the night but does not continue to cool at the same rate as the McGetchin cold spot later in the night. This may suggest a subsurface layering structure that is intermediate between the two models, or thermophysical properties that differ from the modeled assumptions. More broadly, the discrepancy highlights the need for further constraints on subsurface structure, including measurements sensitive to greater depths than Diviner nighttime temperatures, improved laboratory constraints on the relationship between regolith thermal conductivity, density, and other geotechnical properties, and in situ measurements of vertical density structure on future landed missions. Nevertheless, comparative trends between cold spots are likely to persist for different thermal models. Additionally, if reworking depth scales approximately linearly with crater diameter, the observed rollover in cold spot ∆*T*_cor_ is less sensitive to systematic shifts in the density profile due to different thermophysical property assumptions, as the position of the rollover is related to the absolute diurnal skin depth.

### Cumulative effect of cold spot formation on regolith evolution

The scaling of cold spot properties with crater diameter has implications for global regolith evolution. Because of their large spatial extent, a given point on the surface is affected by the formation of a cold spot relatively frequently, on timescales of order ∼10 Ma (*6*). The cumulative effect of cold spot decompaction on regolith evolution may therefore be substantial.

We explore this using a model of cold spot accumulation where craters are stochastically placed on a surface with sizes defined by a power-law production function (PF) (see Materials and Methods). Each crater generates a self-similar cold spot whose peak intensity scales with crater size according to Fig. 5 and decreases radially following the profile of new cold spots (fig. S5). We treat the H- and δ-models as end-members for the depth of subsurface decompaction and track the maximum modification depth experienced at each point on the surface through time. This approach is numerically analogous to analytical impact crater gardening models, which use Poisson statistics to describe crater saturation and the resulting depth of regolith overturn with time (*34**–**36*). Here, gardening refers to excavation and overturn of material within the transient-crater cavity, distinct from the more distal regolith modification represented by cold spots.

**Fig. 7.**
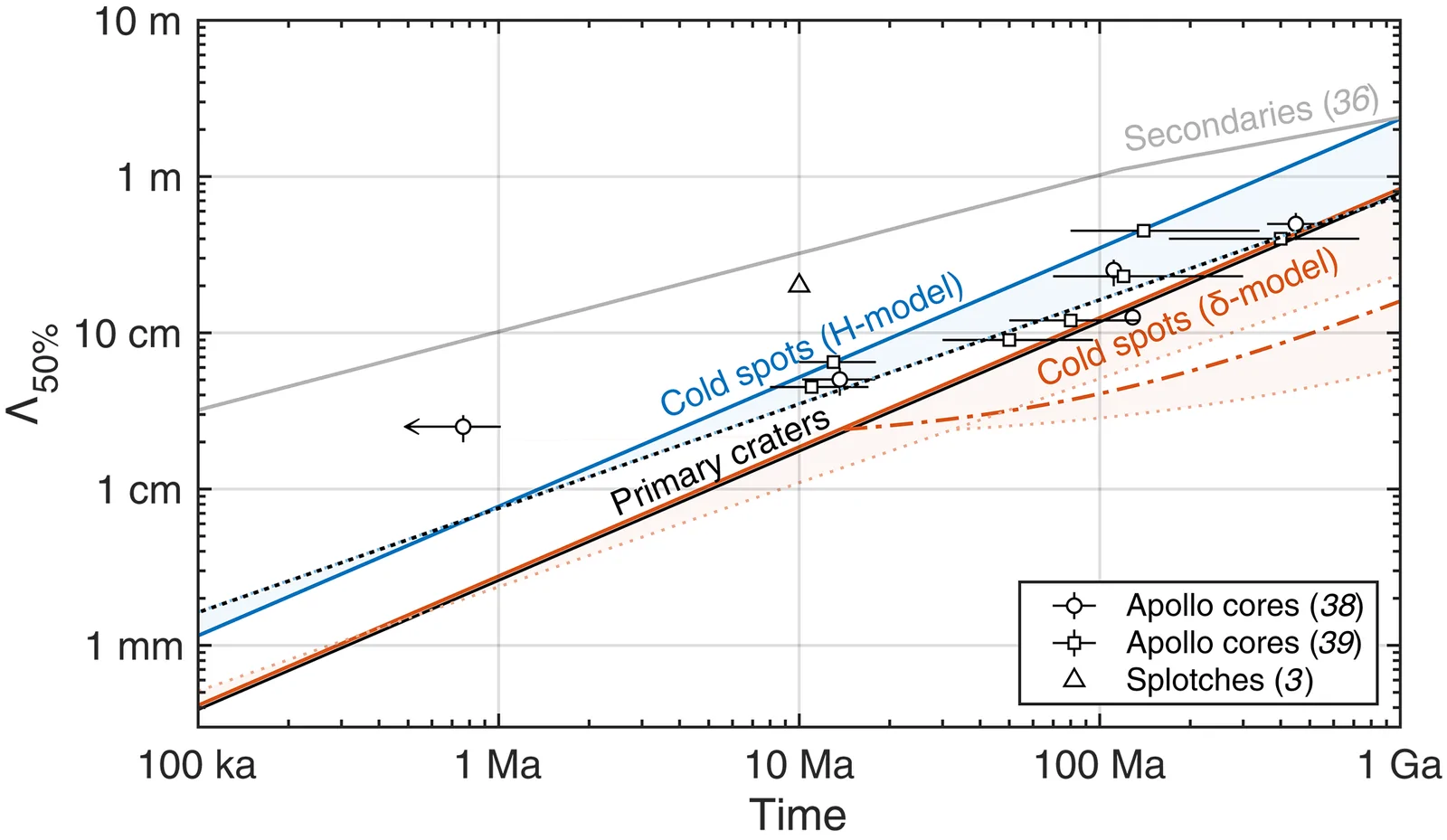
Rate of regolith modification by cold spot formation. Median modification depth versus time for gardening by primary craters (black) and regolith modification from cold spot formation modeled using the H- (blue) and δ-models (red), compared with cosmic-ray exposure ages from Apollo drill cores (*38*, *39*). The dash-dotted red line corresponds to a flattening of the δ-model for δ > 2.4 cm (Fig. 5C). Solid lines show models run using a power-law primary PF fitting the NPF (*5*) and WPF (*37*) between 0.01 and 1 km. Dotted lines show the same models run with a slightly steeper primary PF (fig. S6), possibly suggested by new crater production (*4*). Shaded regions highlight the sensitivity of the cold spot modification rate to the primary PF; shading between the primary-crater models is omitted for clarity.

The solid lines in Fig. 7 show the predicted median depth of regolith modification from cold spot formation through time, assuming a power-law primary PF consistent with the Neukum PF (*5*) and Williams PF (*37*) between 10 m and 1 km diameter (fig. S6) (see Materials and Methods). The δ- and H-models predict that a median point on the surface is affected by a cold spot from a crater >30 m roughly once every ∼10 Ma, producing decompaction to depths of ∼2 to 6 cm, respectively. This range is comparable to predicted overturn depths from impact gardening by primary craters, which falls near the δ-models and below the H-model by a factor of ∼3. Cosmic ray exposure ages obtained from Apollo cores also generally fall within the modeled range (*38*, *39*), although the models do not capture the more rapid reworking inferred for the upper ∼2 cm. In the case where δ scaling flattens for larger craters, the rate of regolith modification decreases after ∼13 Ma, leading to an order-of-magnitude difference between models by 1 Ga. However, this discrepancy does not affect modification on shorter timescales, where the cold spots of smaller craters are most relevant for geometric saturation.

Both modeled primary-crater gardening and distal modification from cold spot formation fall below predictions for gardening by secondary craters (*35*, *36*). This is notable because one proposed mechanism for cold spot decompaction is secondary cratering and related processes. One explanation is that secondary impacts may garden the regolith without producing decompaction detectable by Diviner. In this case, cold spots do not record all, or even most, of the centimeter-scale reworking by secondaries. The DLRZs around new craters indicate surface alteration over distances much greater than the cold spot itself. We interpret these regions as roughening at submillimeter scales, but they may be modified to greater depth. However, any deeper modification throughout the DLRZs, if present, would have to occur without decompacting the regolith enough to affect nighttime temperatures; otherwise, cold spots would extend to comparable distances. Alternatively, the rate of secondary gardening may be overestimated. One possible explanation is that secondaries are strongly clustered around their source primaries rather than uniformly distributed globally. As a result, the number of secondaries affecting a median point on the surface may be substantially lower than the global mean (*40*).

The balance between cold spot modification and primary-crater overturn also depends on the shape of the primary crater PF. Because cold spots require larger craters than primary overturn to achieve the same regolith modification depth, a steeper PF, with a greater proportion of small craters relative to large ones, causes primary overturn to outpace cold spot modification. Detections of new craters on the Moon may suggest a slightly steeper contemporary PF (*3*, *4*): Counts generally agree with the NPF (*5*) at ∼10 m, but fewer 20- to 50-m craters are observed than predicted, although the sample size remains small. To test the sensitivity of cold spot modification rates to the primary PF, we consider a slightly steeper PF that approximates estimates of new crater production (fig. S6) (*4*). We adopt a PF power-law slope of *−*3.5, compared with ∼−3.21 for the NPF and WPF over the 0.01- to 1-km size range (*5*, *37*). In this case, cold spot reworking is reduced relative to primary gardening, such that the H-model is roughly comparable to primary gardening and the δ-model falls below it. This sensitivity highlights the importance of continued monitoring of new crater production to accurately constrain the primary cratering rate. Nevertheless, under the range of assumptions considered here, distal regolith modification by cold spots remains within a factor of a few of primary cratering, making cold spots an important agent of lunar regolith evolution.

## DISCUSSION

The prominent cold spot of McGetchin crater, together with the smaller new craters, shows that cold spot properties scale with crater diameter, indicating progressively deeper regolith modification by larger impacts. Notably, extrapolating the H- and δ-models to larger sizes suggests that a fresh 1-km crater would alter the regolith to depths of 38 cm to 2.2 m in the most prominent portions of the cold spot, or ∼8.4 cm if the δ-model rate of subsurface modification flattens for larger craters. This implies that larger craters may decompact the regolith to depths greater than can be inferred from nighttime surface temperatures alone, which are sensitive only to material properties within a few multiples of the diurnal thermal skin depth.

Cold spot decompaction does not necessarily require overturn of the regolith, and the degree and nature of mixing likely depend on the formation mechanism. Several mechanisms have been proposed, including (i) a granular flow initiated by a cascade of secondary impacts, similar to ballistic sedimentation (*6*); (ii) an impact-generated gas flow (*6*); and (iii) seismic shaking that lofts and dilates the upper regolith (*41*, *42*). McGetchin crater provides a clear opportunity to distinguish among these mechanisms because its cold spot structure is well resolved and pre- and post-impact images reveal subtle surface changes not detectable for preexisting cold spots.

The spatial similarity of the cold spot thermal signature, proximal ejecta pattern, and DLRZ [figures 1 and 6 in (*1*)] is suggestive of an ejecta-related process. Additionally, the increasing extent of the cold spot, daytime EPF anomaly, and DLRZ, consistent with their expected sensitivity to progressively finer-scale disturbance, supports a common formation mechanism and a continuum of surface modification. Traditional ballistic sedimentation by a series of secondary and tertiary impact events (*6*, *43*, *44*) or the initiation of a granular flow (*6*) implies mixing of the regolith within the affected layer. By contrast, seismic shaking can inflate surface material, but may not require complete overturn within the affected layer. While seismic shaking can produce ray-like structures through interactions with subsurface heterogeneity (*42*), it is unlikely that these rays would correspond to the visible ejecta pattern seen in the proximal ejecta and DLRZ. However, seismic shaking from the re-impact of ejected material may contribute to decompaction along cold spot rays. Other ejecta-related mechanisms, such as lateral shearing of the subsurface by ejecta (*45*), may plausibly alter the regolith without requiring complete mixing. Further investigation of cold spot nighttime cooling curves and fading behavior may provide additional insight into the formation mechanism.

Regardless of mechanism, we predict that repeat cold spot formation decompacts the shallow regolith at a rate comparable to overturn by primary craters. This is especially notable in the context of the Moon’s vertical regolith structure, where a thin layer of decompacted regolith overlies more compacted material at depth. Thermal mapping and landed measurements suggest that this layering is largely uniform across the Moon at ∼100-m lateral scales (*25*–*27*), despite local variability within individual sites (*46*). This widespread uniformity implies a steady-state balance between processes that decompact and recompact the regolith, such as impact cratering, vibrational settling (*33*), and thermal cycling (*47*), which is reached on relatively short timescales. Decompaction from cold spot formation may play a role in maintaining the stratified regolith structure observed across much of the Moon. The estimated decompaction depth is likely intermediate between the bounds set by our end-member thermal models, which represent a gradual density change over a relatively large thickness (H-model) or an abrupt decompaction over a smaller thickness (δ-model). Consistent with this interpretation, the nighttime cooling curve of the McGetchin cold spot is also intermediate between the two models. This mismatch may reflect an incorrect representation of the near-surface density profile, uncertainties in the assumed thermophysical properties, or both. These inferred modification depths should be interpreted as effective values that assume laterally homogeneous properties within the cold spot. Unresolved heterogeneity is likely, as larger cold spots, such as the one associated with Bandfield crater (90.76°E, −5.39°N, 898-m diameter), show patchy temperature variations that often correspond to visible surface disturbances (*8*). The regolith structure inferred from thermal modeling may reflect an areal mixture of thermophysical properties, which may differ from the modification depth inferred for a single homogeneous regolith column. Further study of cold spot cooling curves, and how those curves evolve as cold spots fade, could provide new insight into the vertical structure of cold spot regolith.

This work also has implications for other regolith-covered bodies. Similar low-thermal inertia features have been observed around young craters on Mars (*12*), and cold spots have been predicted to form on Mercury (*48*) and may be detectable by the MERTIS instrument on BepiColombo (*49*). Like the Moon, change detection in MESSENGER images has revealed several contemporary alterations on Mercury (*50*), some of which exhibit ray-like albedo anomalies and are likely impact craters, which may host cold spots. Cold spot formation may therefore represent a broader signature of impact-driven regolith modification and a notable agent of surface evolution on regolith-covered bodies across the solar system.

## MATERIALS AND METHODS

### Diviner nighttime temperatures of new craters

Table S1 shows the location, diameter, and constraints on formation date for the largest 21 new craters with diameters >20 m, determined using change detection in LROC images (*1*, *3*, *4*). For each new crater, we compile Diviner channel 6–8 (13 to 23, 25 to 41, and 50 to 100 μm) brightness temperature data pre- and post-impact for 0.5° × 0.5° regions centered on the crater and gridded at 128 pixels per degree (ppd). Following the approach in (*27*), we produce bolometric temperature, *T*_bol_, maps interpolated to local midnight. A thermophysical model that accounts for the effects of scattering, re-emission, and shadowing from surrounding topography (*27*) is used to determine the temperature anomaly, ∆*T*_bol_, corrected for topography. For each crater, we bin ∆*T*_bol_ radially in 240-m annuli and characterize the cold spot, if present, by the peak median temperature anomaly. Most of the new cold spots were analyzed using maps of temperature difference between pre- and post-impact. However, for craters that formed shortly after the start of the LRO mission and which do not have sufficient pre-impact nighttime temperature coverage, we instead use the difference between post-impact data and thermal model predictions.

McGetchin crater has been observed by Diviner during nine targeting campaigns since its discovery, with each campaign including several targeted observations acquired across staggered consecutive orbits. As of 18 February 2026, the full dataset consists of 43 orbits. Off-nadir pointing during these observations produced emission angles ranging from 0° to ∼60° (fig. S1). Diviner off-nadir EPF (*22*) observations show substantial variation in measured *T*_b_ with viewing angle. Nighttime *T*_b_ decreases with increasing emission angle, an effect attributed to surface roughness (*17*). We apply an empirical emission angle correction derived using Diviner nighttime EPF data to adjust off-nadir measurements to equivalent nadir *T*_b_ (*27*): ε_app_ = cos(θ)^0.151^ where ε_app_ is apparent emissivity relative to nadir and θ is emission angle (<60°). A composite bolometric temperature map gridded at 128 ppd is produced using an empirical function to interpolate the nighttime cooling curve within each pixel to local midnight, as in (*27*). Low emission angle data are prioritized when present. Moderate emission angle data are used for pixels where there is insufficient low emission angle data. This mostly occurs in the edges of the composite swath, where higher emission angles widen the swath width of Diviner observations.

Inspection of consecutive off-nadir orbits also revealed a potential offset in Diviner pointing. The apparent position of McGetchin crater and other features shifts north in east-looking orbits and south in west-looking orbits. We find that this offset can be corrected by applying a constant adjustment to the Diviner azimuth actuator position of −0.4°. Similar pointing errors have previously been identified and corrected in the elevation actuator position (*27*), but azimuth offsets are more difficult to detect because they become apparent only in high–emission angle off-nadir data. While a constant offset is sufficient to correct the post-impact McGetchin crater dataset, further work is needed to determine whether this offset has varied over the course of the mission. This offset does not affect standard nadir-pointing observations.

### Effect of Diviner resolution on cold spot recovery

Cold spots formed by small craters (∼30 to 50 m) are expected to approach the spatial resolution of Diviner. As a result, the peak temperature anomaly measured by Diviner will be lower than the true peak value: Contributions from less anomalous surrounding terrain within the instrument field-of-view will mix with the cold spot signature and result in lower temperature contrasts. To account for this resolution effect, we simulate idealized cold spots with varied crater diameter and a self-similar radial ∆*T*_bol_ profile based on the McGetchin crater cold spot (Fig. 4D). We then convolve the bolometric radiance with the Diviner spatial response function (SRF) (*27*, *29*) to determine a recovery factor, defined as the ratio between the Diviner-measured and true peak ∆*T*_bol_ (fig. S3A). The recovery factor does not change meaningfully for typical cold spot temperature anomalies of −1 to −10 K, because over that range, the *T*^4^ radiance relation is effectively linear around the ∼100 K background temperature. This modeling indicates that peak ∆*T*_bol_ is recovered to >90% of its true value for craters larger than ∼100 m, but declines to ∼50% for ∼25-m craters. We use these results to calculate the resolution-corrected peak temperature anomaly, ∆*T*_cor_, for each new crater (table S1).

### Cold spot catalog

In this work, we compare new cold spots with preexisting cold spots. Bandfield *et al.* (*6*) and Williams *et al.* (*9*) created a global catalog of ∼2000 cold spots between ±50°N latitude. However, recent maps with corrections for topography and improvements in data georeferencing reveal more faint and small cold spots than were previously detected (*26*, *27*). We use the Global High-Resolution Mosaic (GHRM) bolometric temperature anomaly, ∆*T*_bol_, map (*27*) to catalog additional cold spots not identified in the original survey. This is an ongoing effort, and the current updated survey includes an equatorial swath from −10°N to 0°N with improved identification of small and faint cold spots. In addition, we update the original global cold spot catalog by refining crater coordinates using NAC images and add several additional cold spots (fig. S4). Although this updated catalog is not yet globally complete, it provides a representative sample of cold spot properties across all lunar terrains sufficient for comparison with newly formed cold spots. Craters were grouped as either highlands or mare according to the mare boundaries determined by (*51*). The peak temperature anomaly for each cold spot is determined as the peak value of its radially binned ∆*T*_bol_, following the same approach used for the characterization of new cold spots. The peak RA of each crater is determined as the maximum RA value within 5 crater radii, including pixels containing the crater interior, taken from the GHRM RA map (*27*).

### Cold spot accumulation model

The cumulative effect of cold spots on subsurface modification can be quantified using an approach analogous to impact gardening models. Craters are produced following a PF that can be approximated by a power law

$$N={\mathrm{uD}}^{v}$$ (2)

where *v* is the power-law slope, *u* is a coefficient describing the production rate, and *N* is the number per unit area and time. The cratering rate has changed throughout lunar history, but is considered to have been roughly constant over the last ∼3 Ga (*5*). We consider two primary PFs: (i) a power-law fit to the Neukum PF and Williams PF (*5*, *37*) and (ii) a slightly steeper power law, which may be suggested by estimates of new crater production (*4*).

As craters accumulate, new craters have a progressively increasing probability of overlapping, until the surface is fully saturated by craters of some size. This can be mathematically captured using Poisson statistics and is the basis for impact gardening models (*34**–**36*). If each crater excavates to some depth proportional to its diameter, the reworking depth, Λ, as a function of time is (*36*)

$$\Lambda =d{\left(\frac{4\lambda }{u\pi c^{2}t}\frac{2+v}{v}\right)}^{\frac{1}{v+2}}$$ (3)

where *d* is the ratio of overturned depth-to-diameter and λ describes the expected number of events per interval (e.g., λ ≈ 0.693 corresponds to a 50% probability of an event occurring). In these gardening models, redistribution of material excavated from within the crater cavity is treated statistically, with subsequent events assumed to replace material within previously excavated regions. This equation perfectly describes the accumulation and gardening by cylindrical pucks, with defined diameter and depth. However, craters do not rework to a constant depth across their entire width, but instead excavate material from within a bowl- or parabolic-shaped cavity. Craters are instead approximated as circular caps by defining the fraction of the crater’s width, *c*, that overturns to depth *dD*. Cold spots have a broad and complex radial profile that extends over a large distance, making determination of effective diameter and depth values for this spherical-cap approximation nontrivial.

We create a numerical model that behaves analogously to the analytical model, where craters are placed stochastically on a surface following a power-law size-frequency distribution, and each forms a cold spot with a realistic radial profile of regolith modification. Figure S5 shows the radial profile of the McGetchin (NC-A), NC-B, and NC-I cold spots in terms of temperature anomaly, as well as equivalent *H* and δ. We find that the radial profiles can be reasonably well fit to an empirical function similar to the Morse potential function

$$\Delta T=\Delta T_{\text{peak}}\left\{1-{\left[1-e^{-\left(R-R_{\text{peak}}\right)/B}\right]}^{2}\right\}$$ (4)

where *R* is the radial distance in crater radii, *R*_peak_ is the radial position of the cold spot peak anomaly, and *B* describes the scale-width of the radial profile. Because *H* and δ have nonlinear scaling with temperature, the *H* and δ radial profiles drop off differently with distance, requiring different *B* values. Most notably, the δ-model requires a broader profile than the H-model. These profiles are used to produce self-similar cold spots, where the peak intensity scales with crater size according to Fig. 5. The maximum depth of modification is recorded for each pixel as craters accumulate, and the median modification depth is tracked with time. Primary-crater gardening is modeled by assuming that each primary crater forms a transient crater whose diameter is 84% of the final crater diameter, and with a transient depth-to-diameter ratio of 1/3 (*52*). The gardening depth associated with each crater is assumed to be 1/3 of the transient crater depth (*35*). The output of the numerical model is used to determine parameters *d* and *c* in Eq. 3 for each run.
