# Supplementary material for: New lunar crater reveals extensive distal regolith modification
Source: Sci Adv. 2026 Sep 16;12(38):eaeh9568. doi: 10.1126/sciadv.aeh9568 (PMC13580542; doi:10.1126/sciadv.aeh9568)
Supplement: Supplementary file 1 [file sciadv.aeh9568_sm.pdf]

Science Advances
AAAS

Supplementary Materials for

New lunar crater reveals extensive distal regolith modification

Tyler M. Powell *et al.*

Corresponding author: Tyler M. Powell, tyler.powell@jhuapl.edu

Sci. Adv. **12**, eaeh9568 (2026)
DOI: 10.1126/sciadv.aeh9568

This PDF file includes:

Figs. S1 to S6
Table S1

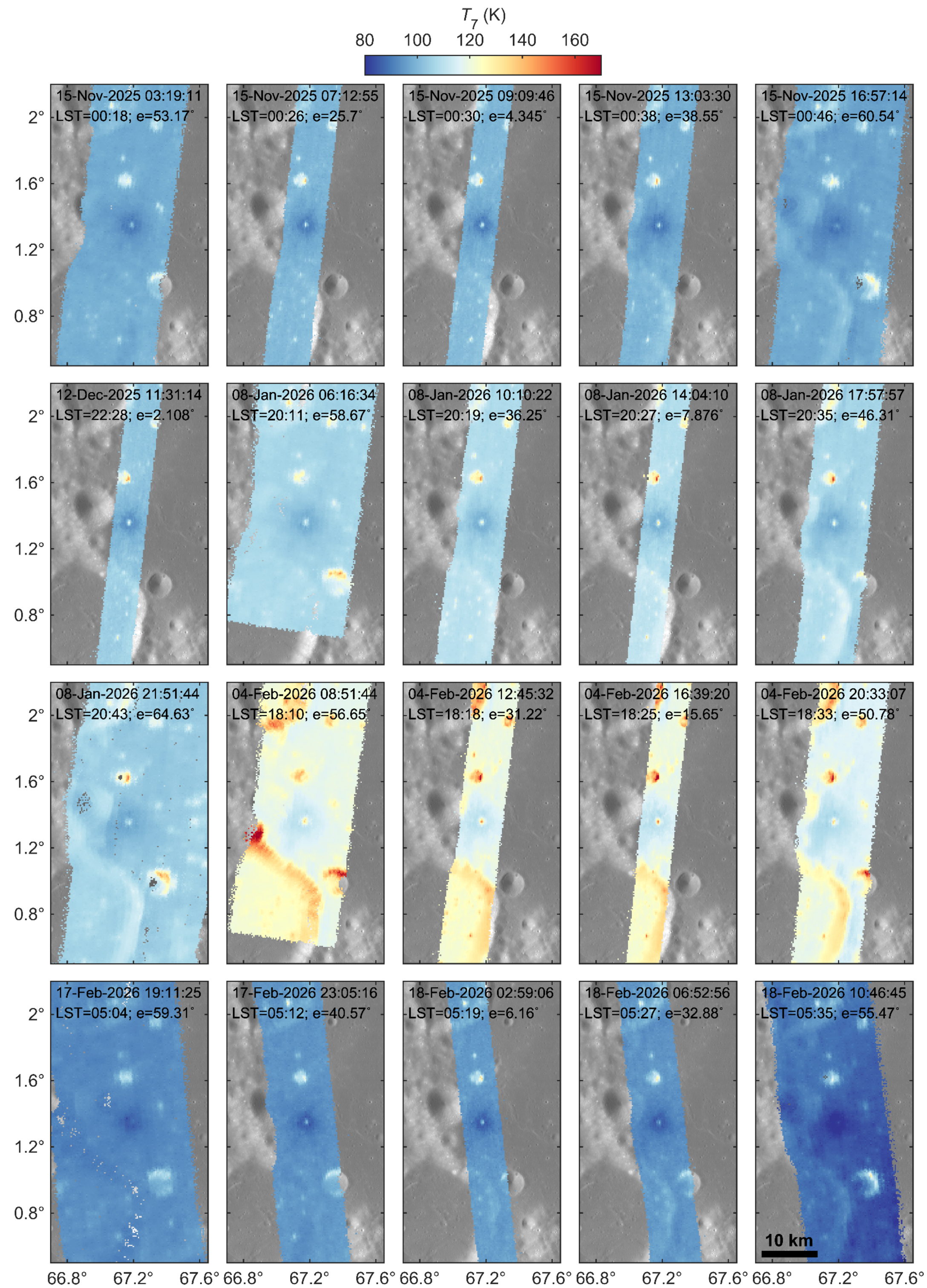

Fig. S1.

Diviner channel 7 nighttime brightness temperature observations of McGetchin crater post-impact. Subpanel captions indicate the observation time in UTC, local solar time (LST), and emission angle (e).

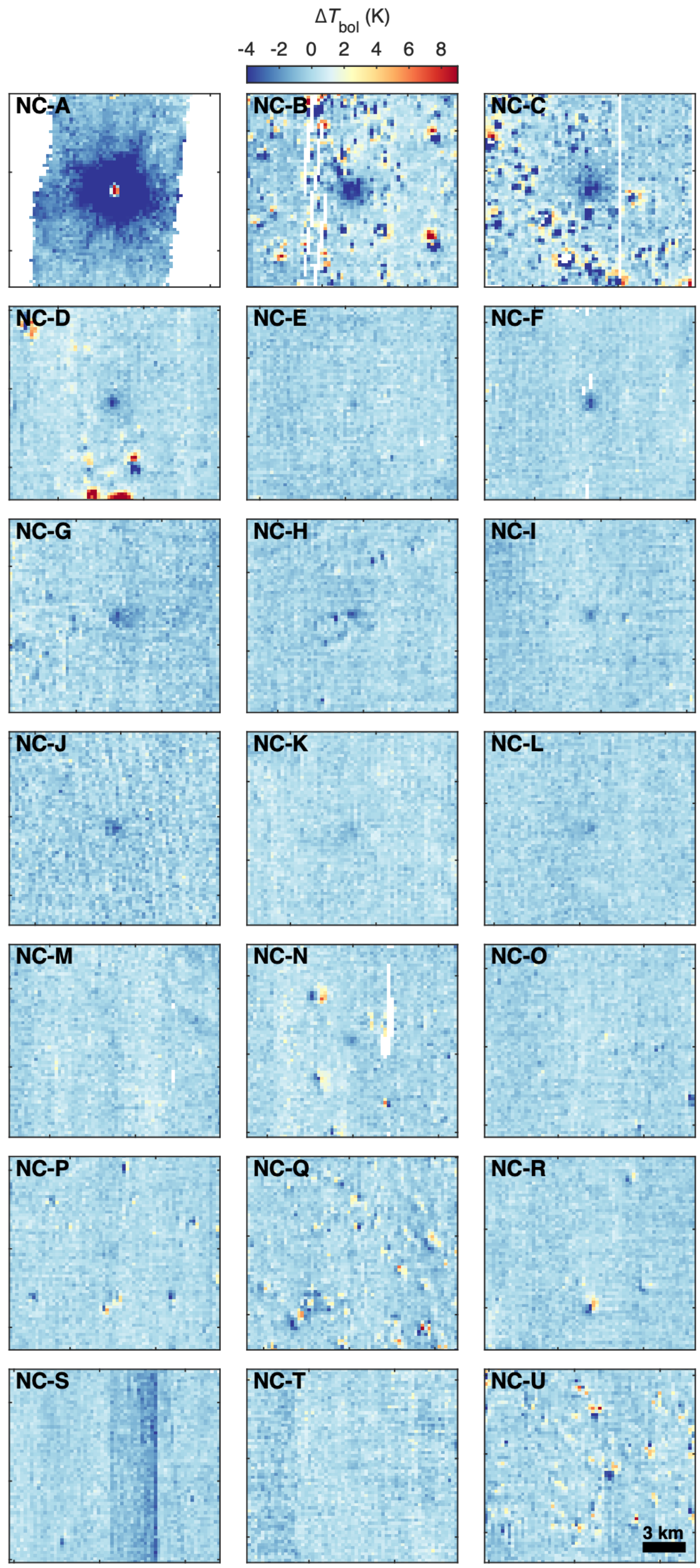

Fig. S2.

Difference between post- and pre-impact bolometric temperature for all 21 newly identified craters in table S1 (*1,3,4*). Panels share the same color and spatial scale for comparison.

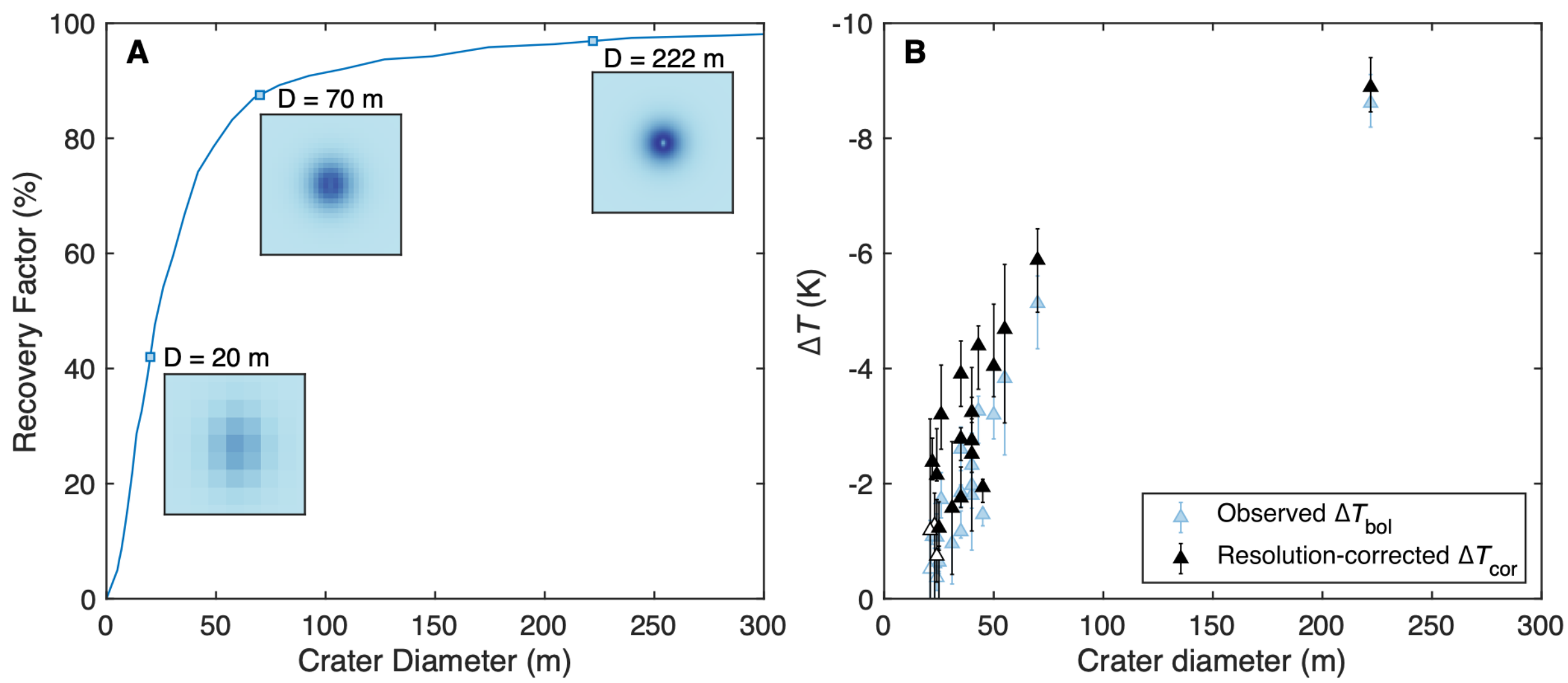

Fig. S3.

A) Recovery factor of peak ΔT_{bol} for self-similar cold spots with varying crater diameter. Each idealized cold spot has a radial profile based on that of the McGetchin cold spot. Bolometric brightness temperatures are convolved with the Diviner spatial response function (SRF) (*27,29*). The resulting radial profiles are binned in 240 m radial bins. B) Observed peak ΔT_{bol} and resolution-corrected ΔT_{cor} of new cold spots, adjusted according to their diameters and modeled recovery factor.

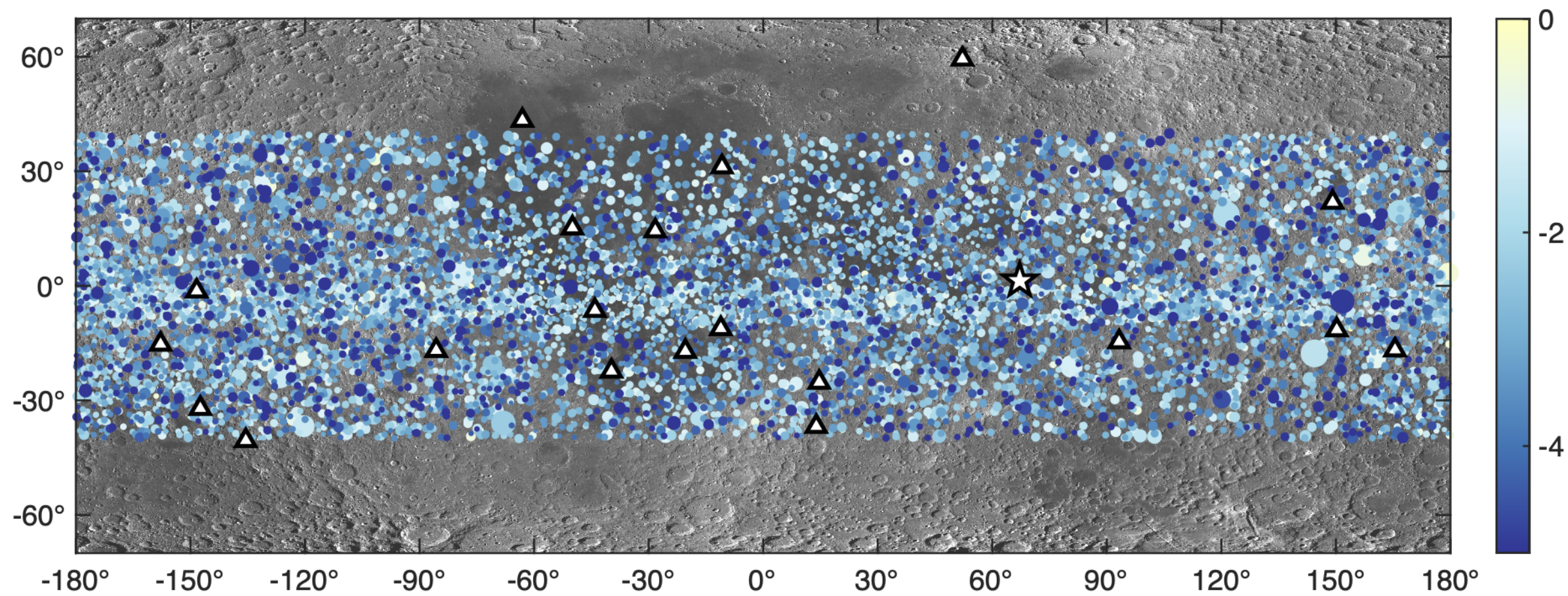

Fig. S4.

Catalog of cold spots from 40˚S to 40˚N latitude with marker sizes scaled to crater diameter. Triangle markers correspond to contemporary craters formed during the LRO mission (*3,4*), and the star marker corresponds to McGetchin crater (*1*).

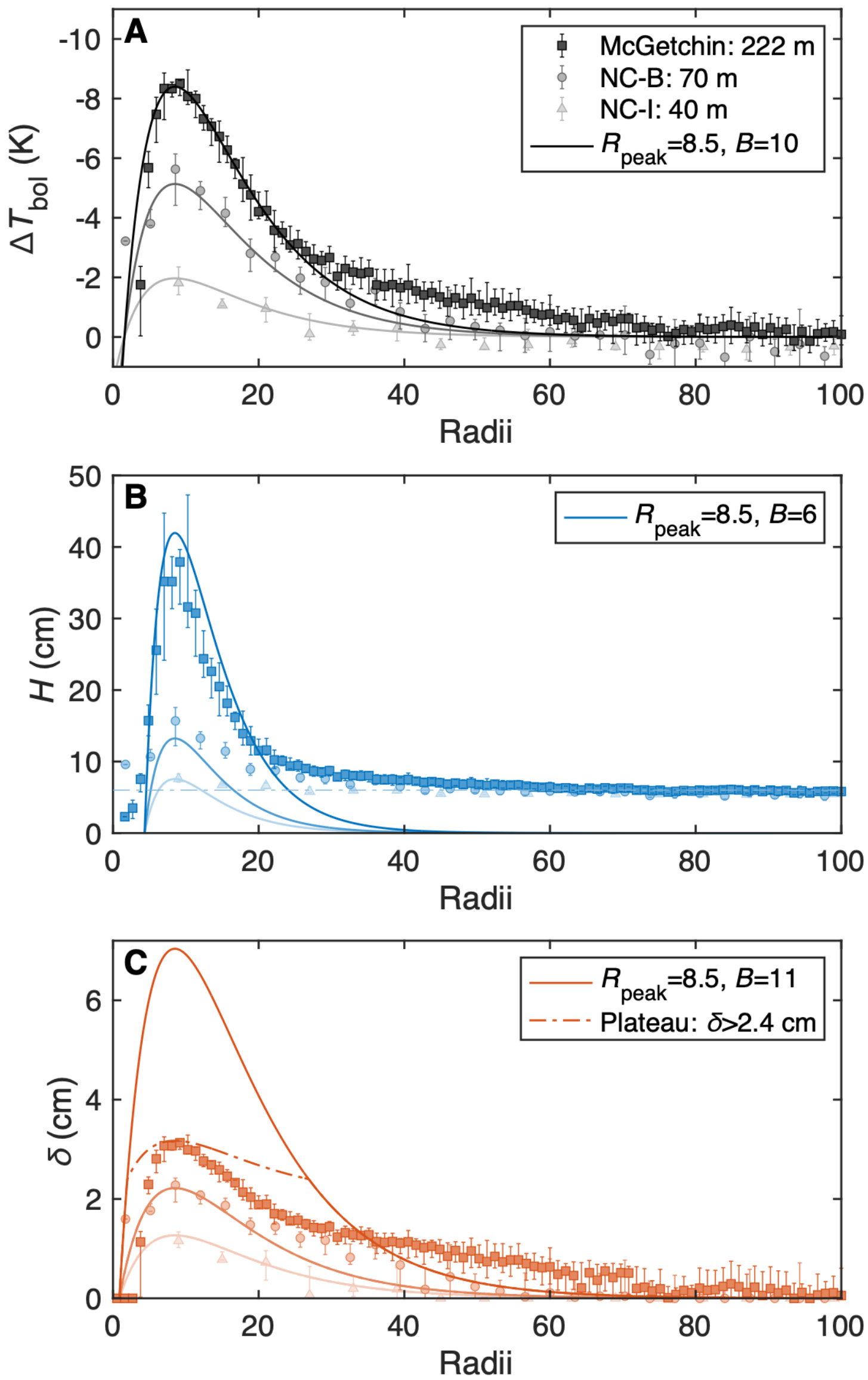

Fig. S5.

Radial profile of A) temperature anomaly, B) H-parameter, and C) δ for the cold spots around McGetchin crater, NC-B, and NC-I. Each is approximated by an empirical function that describes the radial drop-off of properties with distance, where the peak anomaly scales with crater diameter. In C, the dash-dotted line corresponds to a flattening of δ for values >2.4 cm, as shown in Fig. 5C.

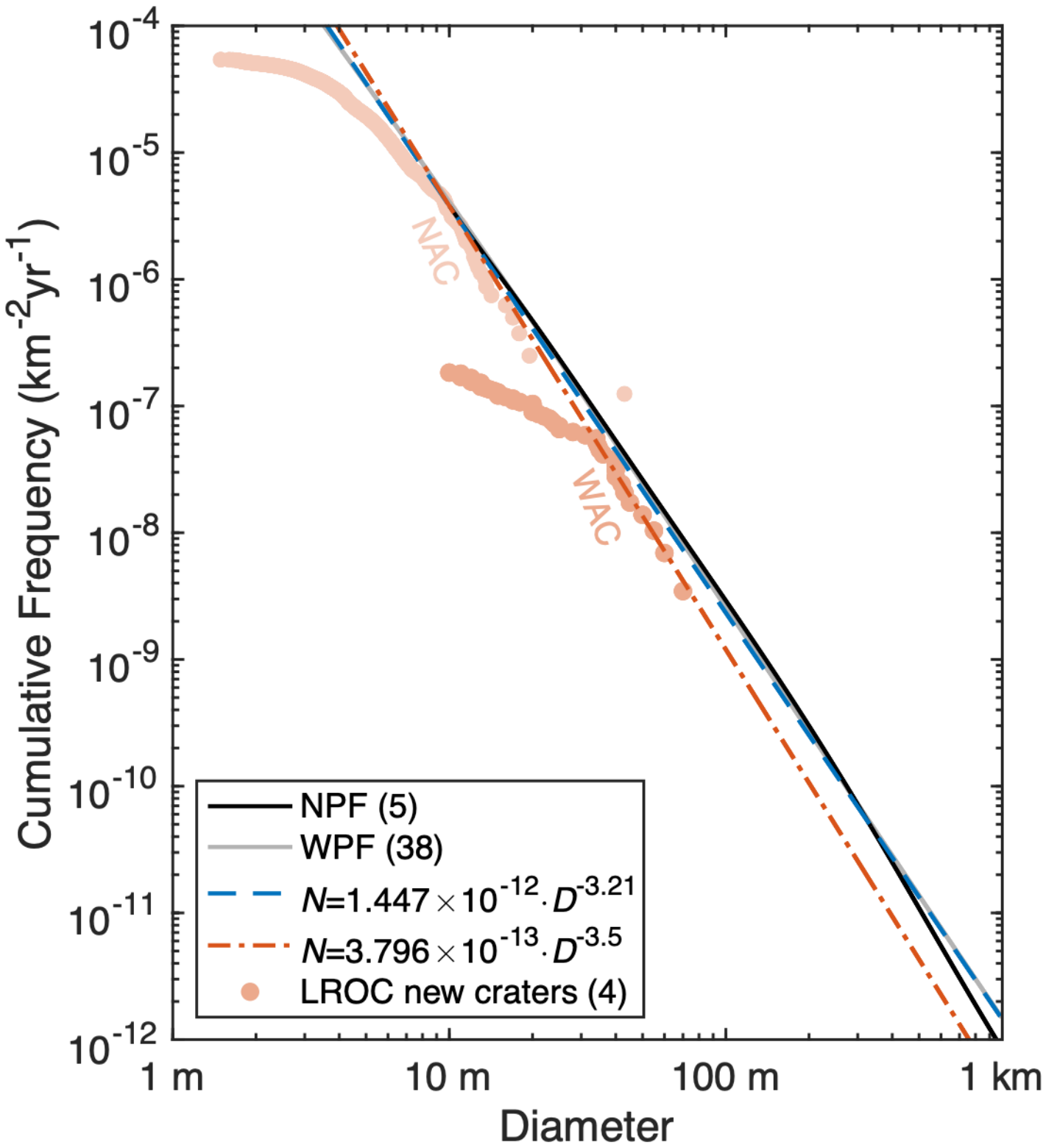

Fig. S6.

Cumulative size-frequency distribution (SFD) of contemporary lunar impact craters, extracted from (*4*), compared with the Neukum (*5*) and Williams (*37*) production functions. The dashed blue line shows a power law fit to the NPF between 0.01 and 1 km. The dashed red line represents a slightly steeper power law approximating estimates of recent crater production. In the power-law fit equations, D is in units of km and N is in units of km^{-2}yr^{-1}.

Table S1.

Summary of new craters and the properties of their cold spots, if present. Formation date estimates were determined by their latest pre-impact non-detection and earliest post-impact detection in either Diviner or LROC images.

Index	Longitude	Latitude	Diameter (m)	Formation date min.	Formation date max.	ΔT_{bol} (K)	ΔT_{cor} (K)	H_{cor} (cm)	δ_{cor} (cm)
A[a]	67.1765°	1.3536°	222	2024-04-11	2024-05-22	-8.611	-8.889	45.46	3.262
B	-39.730°	-22.352°	70	2012-10-02	2012-10-29	-5.135	-5.885	16.49	2.351
C	-63.066°	43.350°	55	2010-04-17	2010-08-15	-3.831	-4.683	13.09	2.029
D	-44.068°	-6.436°	50	2013-07-02	2013-08-12	-3.195	-4.041	11.15	1.838
E	150.216°	-11.515°	45	2015-09-13	2015-12-17	-1.464	-1.936	7.723	1.199
F	-28.271°	14.395°	43	2013-12-12	2014-01-08	-3.263	-4.398	12.04	1.941
G	-135.510°	-40.526°	40	2016-08-27	2016-10-20	-1.802	-2.515	8.494	1.391
H	-147.426°	-32.110°	40	2014-02-26	2014-04-22	-2.321	-3.240	9.637	1.603
I	-157.826°	-15.289°	40	2016-02-06	2016-03-31	-1.969	-2.748	8.849	1.465
J	52.186°	59.370°	35	2015-10-14	2016-01-21	-2.602	-3.907	11.15	1.822
K	149.145°	21.881°	35	2013-03-28	2013-07-14	-1.168	-1.753	7.543	1.144
L	93.224°	-14.664°	35	2015-09-17	2015-11-25	-1.849	-2.777	8.893	1.473
M	-85.649°	-16.933°	31	2013-01-09	2013-02-05	-0.960	-1.576	7.299	1.084
N	-20.402°	-17.166°	26	2013-09-11	2013-09-11	-1.727	-3.198	9.564	1.590
O	165.474°	-16.785°	25	2017-02-24	2017-08-20	0.136[b]	0.261[b]	5.520[b]	-
P	-11.096°	-11.211°	25	2017-03-09	2017-04-05	-0.639	-1.230	6.911	0.898
Q	-10.793°	31.156°	24	2018-07-26	2018-12-22	-1.073	-2.150	8.102	1.275
R	14.675°	-25.261°	24	2014-02-14	2014-04-10	-0.371[b]	-0.744[b]	6.408[b]	0.622[b]
S	-148.365°	-1.361°	23	2013-11-23	2014-01-03	-0.610[b]	-1.275[b]	6.957[b]	0.933[b]
T	13.901°	-36.819°	22	2014-04-10	2014-05-07	-1.086	-2.374	8.305	1.346
U	-49.964°	15.170°	21	2015-10-25	2015-12-05	-0.517[b]	-1.185[b]	6.874[b]	0.864[b]

[a] McGetchin crater, [b] No cold spot detected
